# Systematic Review and Recommendations to Combine Newer Therapies With Conventional Therapy in Psoriatic Disease

**DOI:** 10.3389/fmed.2021.696597

**Published:** 2021-08-19

**Authors:** Sandeep Arora, Pankaj Das, Gulhima Arora

**Affiliations:** ^1^Department of Dermatology, Army College of Medical Sciences & Base Hospital Delhi Cantt, New Delhi, India; ^2^Consultant Dermatologist, Mehektagul Dermaclinic, New Delhi, India

**Keywords:** psoriasis, combination (combined) therapy, conventional therapy, biologics, guidelines and recommendations

## Abstract

**Background:** Psoriasis continues to have unmet needs in its management despite introduction of newer molecules. Monotherapy with these newer agents may not achieve therapeutic goals in all cases, hence necessitating their combinations with other molecules. Improved understanding of newer as well as conventional treatment modalities and experiences in their combinations hence necessitates therapeutic guidelines for their use in psoriasis.

**Objective:** To review the combinations of treatments reported in literature and recommendations for their use based on best current evidence in literature.

**Methods:** A literature review of MEDLINE database for studies evaluating combinations of newer therapies with conventional therapies in psoriasis was done. Newer therapies were identified as biologic disease modifying anti rheumatic drugs and other molecules such as apremilast while conventional therapies included methotrexate, cyclosporine, or retinoids, phototherapy and others. The therapeutic guidelines are proposed with the aim to provide evidenced based approach to combine newer and conventional agents in day-to-day psoriasis management.

**Findings:** Combination of acitretin and narrow band ultraviolet B (NB-UVB)/Psoralen with ultraviolet A (PUVA) achieves faster clearance and allows reduction of dose of the latter. A variable outcome is reported of methotrexate with TNF-α inhibitors vs. TNF-α inhibitors alone, although addition of methotrexate appears to reduce immunogenicity of TNF-α inhibitors thereby preventing formation of anti-drug antibodies especially in case of infliximab. While combination of acitretin and PUVA is beneficial, combining TNF-α inhibitors and phototherapy too produces better and faster results but long term risks of Non Melanoma Skin Cancers (NMSCs) may preclude their use together. Combination of cyclosporine and phototherapy is not recommended due to greater chances of NMSCs. Adding phototherapy to Fumaric Acid Esters (FAEs) improves efficacy. Apremilast can be safely combined with available biologic agents in patients with plaque psoriasis or psoriatic arthritis not responding adequately to biologics alone. Hydroxyurea and acitretin may be used together increasing their efficacy and reducing doses of both and hence their adverse effects.

**Conclusion:** Selected clinical scenarios shall benefit from combinations therapies, improving efficacy of both conventional and newer agents and at the same time helping reduce toxicity of higher dosages when used individually.

## Introduction

Psoriasis is a chronic relapsing-remitting inflammatory papulo-squamous disease, which affects ~0.51–11.43% of adults worldwide (1). This immune-mediated disease causes chronic inflammation in milieu which not only affects skin, but also joints, blood vessels, heart, liver, and kidneys (2) as well as metabolic syndrome (3, 4). PsA (Psoriatic Arthritis) may be present in >40% of psoriasis patients leading to joint damage and deformities thereby severely affecting QoL (Quality of Life) and physical functioning (5–7). Early diagnosis and treatment intervention are crucial for optimal patient care (8, 9). The chronic relapsing course of disease with these co-morbidities are associated with increased physical and psychological burden, which leads to impaired Quality of Life (QoL) and depression (10). Mild psoriasis responds to topical therapy while moderate to severe psoriasis may need augmentation with phototherapy or systemic agents. Severe psoriasis may sometimes be refractory to one systemic agents requiring combination with another to maintain remission (11, 12). Combining therapeutic agents holds potential in synergistic action for a better control over disease activity. Moreover, a combination may be needed to reduce adverse effects by allowing reduction of dose despite severe disease. However, combining therapies pose challenges in tolerability, acute and long-term adverse effects in the absence of clear overall guidelines. Conventionally, immunosuppressive and non-biologic disease modifying immune-modulatory drugs such as methotrexate, cyclosporine, retinoids, phototherapy, and others have been used. Management of psoriasis has been revolutionized by biologics which have improved management of psoriasis but aren't panaceas either. Combining newer and conventional therapies provide a tantalizing option for managing psoriasis, to achieve prolonged remission and better Quality of Life (QoL). Although there are numerous Randomized Control Trials (RCTs), case series, case reports, and expert opinions proving efficacy of different combinations in various clinical scenarios, literature is lacking in clear cut guidelines on how and when to combine the newer and conventional therapeutic options. This review aims at analyzing data available from studies with highest quality of evidence i.e., RCTs and generate recommendations for combining newer and conventional therapies in psoriatic disease.

## Materials and Methods

### Protocol Development and Eligibility Criteria

**Figure 1:**
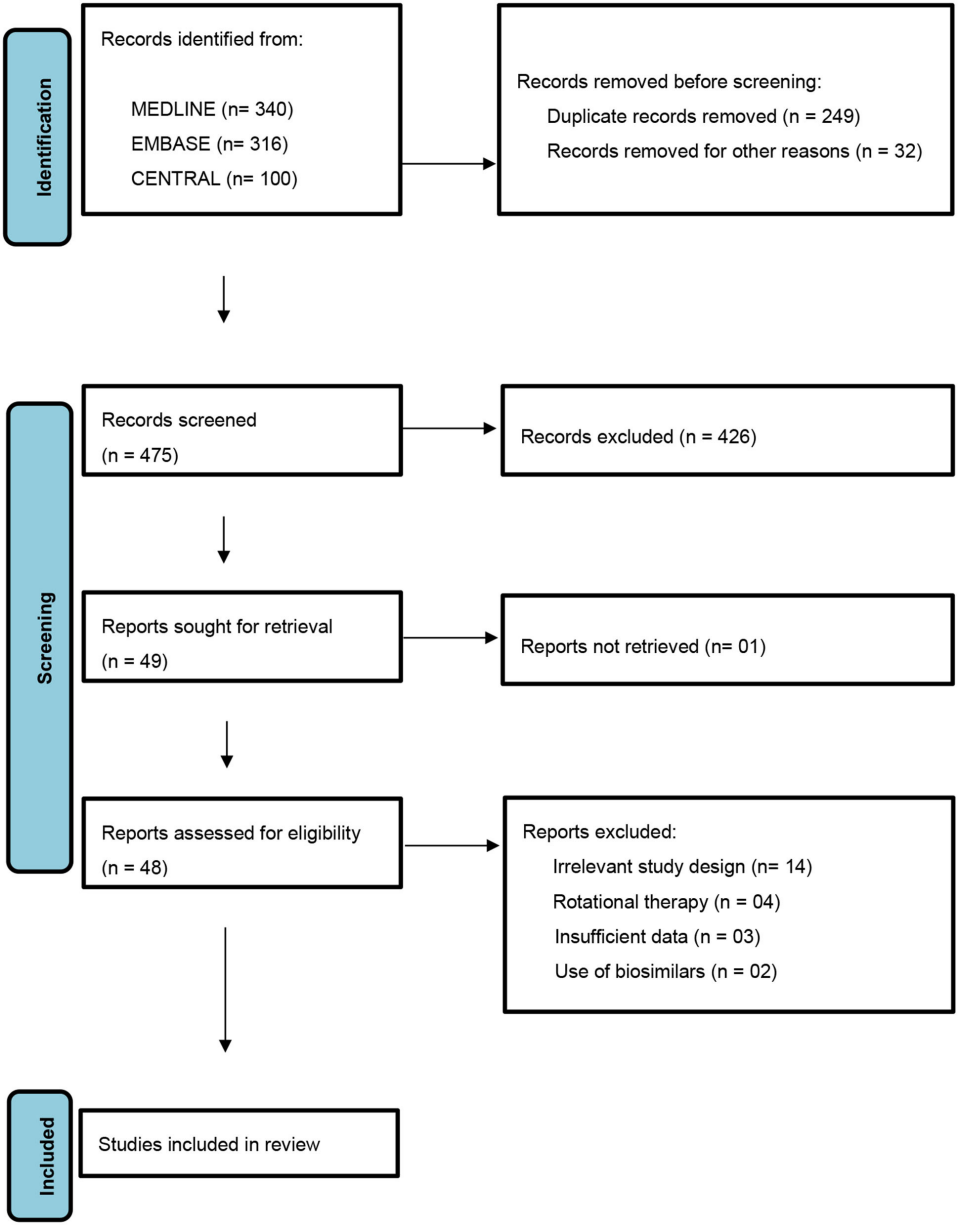
PRISMA flow diagram for selection of studies.

A protocol was designed and followed as laid down by PRISMA (Preferred Reporting Items for Systematic Review and Meta-Analyses) statement (Figure 1). The conventional therapies considered being immunosuppressive and non-biologic disease modifying immune-modulatory drugs such as methotrexate, cyclosporine, retinoids, phototherapy, hydroxyurea, and fumaric acid esters (13). The newer therapies were identified as biologic disease modifying anti-rheumatic drugs namely- TNFα (Tumor Necrosis Factor-α) inhibitors- etanercept, adalimumab, infliximab, golimumab, and certolizumab pegol; IL-17A (Interleukin-17A) inhibitors- secukinumab and ixekizumab; IL-17RA (Interleukin-17 Receptor Antagonist)- brodalumab; IL-12/IL-23 inhibitor- ustekinumab, IL-23 inhibitor- guselkumab; oral PDE-4 (Phosphodiesterase-4) inhibitor- apremilast and tofacitinib selective JAK (Janus Kinase) 1 and 3 inhibitor (14).

### Search Strategy

A literature search was performed for studies conducted in psoriasis therapeutics published before 01 Jan 2021. MEDLINE (OVID, from 1948), EMBASE (OVID, from 1980), Cochrane Central Register of Controlled Trials (CENTRAL), ahead of print subset fraction from Pubmed- not yet published in (OVID MEDLINE), and ongoing trial registries (http://clinicaltrials.gov/) were searched with no language restrictions. The search was carried out through use of keywords targeting all drugs used in conventional as well as newer therapies. In MEDLINE and EMBASE, a methodologic filter for search was used to identify RCTs and clinical controlled trials in Medical Subject Headings and titles and abstracts (adapted from the Cochrane Central Register of Controlled Trials). For potential drug combinations where RCTs were not found, the search was extended to include lower tiers of hierarchy of evidence up to case series. A systematic method in search was used for each database to broaden the search through inclusion of pertinent search terms as relevant citations were recognized (i.e., by scrutinizing references and citing articles).

### Search Terms

The search terms which were used are as follows: (“methotrexate” OR “cyclosporine” OR “ciclosporine” OR “acitretin” OR “phototherapy” OR “hydroxyurea” OR “fumaric acid esters” OR “conventional” [MeSH term] OR “drugs” [MeSH term] OR “etanercept” OR “infliximab” OR “adalimumab” OR “secukinumab” OR “golimumab” OR “ixekizumab” OR “ustekinumab” OR “guselkumab” OR “certolizumab pegol” OR “apremilast” OR “tofacitinib” OR “biologics” [MeSH term] OR “psoriasis” [MeSH term] OR “combination” [MeSH term] OR “therapy” [MeSH term]).

### Inclusion Criteria

Randomized Controlled Trials (*N* > 10) which reported on the efficacy and safety of combined use of conventional and newer drugs in psoriatic disease were included. Potential combinations in which RCTs have not been carried out, studies with lower levels of evidence were also included.

### Exclusion Criteria

*In-vitro*, preclinical and animal studies, case reports and expert opinions were excluded from the review as well as all studies not meeting the inclusion criteria. Studies with rotational or sequential therapies using these drugs as well as those combining alternative medicines (i.e., Chinese herbal) were excluded. Biosimilars were excluded from the study to maintain uniformity on drug efficacy data.

### Selection of Studies

Using the above keywords, the titles and abstracts from electronic literature search were screened, and full text of articles that met the pre-defined inclusion criteria were obtained. Successively, articles were scanned for inclusion or exclusion. The selection of studies were implemented by 2 reviewers independent of each other (S.A. and P.D.). The quality of each included articles was assessed in agreement with the Cochrane handbook of systematic reviews of interventions 5.1.0 (updated March 2011). Any disagreements between the two reviewers were resolved by commonly drawn consensus by discussion or intervention by a third reviewer (G.A.).

### Data Extraction

Information on the year of publication, study design, study reference, number of patients (N), baseline disease severity, treatment schedule, duration of combination therapy, and period of follow-up were extracted. Critical as well as important outcomes were carefully chosen to determine the quality of evidence. Critical outcomes were defined as the proportion of patients who attained a PASI (Psoriasis Area and Severity Index) of 90, PASI of 75, and a PGA (Physician Global Assessment) of clear or almost clear; discontinuation of a particular drug because of AEs (Adverse Effects); proportion of patients who encountered SAEs (Serious Adverse Events); and mean change in DLQI (Dermatology Life Quality Index). Important outcomes were defined as lack of efficacy leading to withdrawals (number), proportion of patients with AEs (not leading to drug withdrawal), mean time to clearance, mean change in PASI (0–72, 0–18, and 0–16) and mean time to relapse.

## Results

Our literature search yielded 25 RCTs (Table 1) combining different drugs which met the criteria to be included for analysis in the present study. Potential drug combinations for which RCTs have not been done, studies with lesser levels of evidence up to case series were searched for to look for evidence and gaps in research (Table 2).

**Table 1:** Randomized controlled trials which met the inclusion criteria and selected in this study (*n* = 25).

**S no**.	**References**	**Study design**	**No of patients**	**Baseline disease severity**	**Intervention**	**Control group(s)**	**Study length (weeks)**	**Follow up (weeks)**	**Outcome measures used in study analysis**		**LoE**
									**Efficacy**	**Safety**	
1.	Tanew et al. (15)	Randomized double blinded trial	60	≥20% BSA or PASI ≥10	Acitretin 1 mg per kg per day plus four PUVA exposures per week	Placebo plus four PUVA exposures per week	Until complete clearance/maximum of 11 weeks.	11	Complete remission or marked improvement i.e.at least 90% clearing of psoriasis	Percentage of patients with AEs and SAEs/withdrawal because of AEs	2b
2.	Lowe et al. (16)	Randomized controlled trial	37	Moderate to severe chronic plaque type psoriasis	Acitretin 50 mg per day plus UVB	Placebo plus UVB	12	12	Mean PASI at the end of 12 weeks	Percentage of patients with AEs	2b
3.	Lynde et al. (17)	Single- blinded randomized controlled trial	99	≥10% BSA or PASI ≥10	Etanercept 50 mg once a week plus thrice weekly NB-UVB	Etanercept 50 mg once a week	24	24	PASI 90, PASI 75, PGA- clear, minimal, mild, moderate, severe, very severe BSA and DLQI	AEs, SAEs infectious adverse events and injection-site reactions	1b
4.	Park et al. (18)	Randomized, ‘head-to-head' pilot trial	30	≥10% BSA or PASI ≥10 with BMI of 30 or greater	Etanercept induction dose at 50 mg twice weekly for 12 weeks followed by combination of etanercept at maintenance dose of 50 mg weekly with NB-UVB thrice weekly	Etanercept induction dose at 50 mg twice weekly for 12 weeks followed by etanercept monotherapy at maintenance dose of 50 mg weekly	24	24	(i) PASI 75 response after 12 weeks of combination etanercept and NB-UVB therapy (ii)Improvement in average PASI, (iii)Improvement in BSA and (iv) Improvement in PGA	Serious Adverse Events (SAEs) at weeks 12 and 24.	1b
5.	Calzavara-Pinton et al. (19)	Randomized controlled intra-individual trial	20	PASI ≥10, Patients on etanercept alone who did not achieve PASI 75 within 12 weeks	Etanercept at 50 mg twice weekly plus NB-UVB thrice weekly on a selected psoriatic plaque	Covered plaque served as non-irradiated control	24	24	Mean PASI reduction, PASI 90, PASI 75	Percentage of patients with AEs	2b
6.	Gambichler et al. (20)	Randomized controlled intra-individual trial	14	PASI ≥10	Etanercept at 50 mg twice weekly plus NB-UVB thrice weekly on a selected psoriatic plaque	Covered plaque served as non-irradiated control	6	6	Modified PASI reduction, performance of skin biopsies	Percentage of patients with AEs	2b
7.	Wolf et al. (21)	Open-label randomized trial	10	PASI ≥10	Ustekinumab at 45 or 90 mg at week 0 and 4 and plus NB- thrice weekly	Ustekinumab at 45 or 90 mg at week 0 and 4	6	12	PASI of 75, mean change in PASI	Percentage of patients with AEs, withdrawal because of AEs	2b
8.	Mahajan et al. (22)	Randomized, single blinded, placebo controlled trial	40	≥10% BSA	Methotrexate at 0.5 mg per kg once weekly to a maximum of 30 mg per week plus NB-UVB thrice weekly	Placebo plus NB-UVB thrice weekly	24	24	PASI 75, PASI 50	Percentage of patients with AEs and SAEs/withdrawal because of AEs.	2b
9.	Asawanonda et al. (23)	Open-label randomized trial	24	≥20% BSA	Methotrexate at 15 mg per week plus NB-UVB thrice weekly	Placebo plus NB-UVB thrice weekly	24	24	PASI 90, PASI 50, Dermatology Life Quality Index (DLQI)	Percentage of patients with AEs and SAEs	1b
10.	Al-Hamamy et al. (24)	Open-label randomized trial	120	≥10% BSA	Methotrexate at 0.2 mg per kg weekly with a maximum of 20 mg per week plus NB-UVB thrice weekly	(i) Methotrexate at 0.2 mg per kg weekly with a maximum of 20 mg per week (ii) NB-UVB thrice weekly	24	48	PASI 90, PASI 50	Percentage of patients with AEs and SAEs	2b
11.	Zachariae et al. (25)	Open-label randomized trial	60	≥10% BSA or PASI ≥8	Etanercept 50 mg twice weekly for 12 weeks, and then 25 mg twice weekly for 12 weeks plus continued methotrexate therapy	Etanercept 50 mg twice weekly for 12 weeks, and then 25 mg twice weekly for 12 weeks plus methotrexate tapered and discontinued during the 4 weeks	24	24	Physician's Global Assessment (PGA), PASI 50, PASI 75, PASI 90, DLQI.	Percentage of patients with AEs and SAEs/withdrawal because of AEs	2b
12.	Gottlieb et al. (26)	Randomized, double-blind, placebo controlled trial	478	≥10% BSA or PASI ≥10	Etanercept 50 mg subcutaneously twice weekly for 12 weeks followed by 50 mg once weekly for 12 weeks plus methotrexate titrated from 7.5 mg to maximum of 15 mg or tolerated dose.	Etanercept 50 mg subcutaneously twice weekly for 12 weeks followed by 50 mg once weekly for 12 weeks plus placebo	24	24	PASI 90, PASI 75, PASI 50, static Physician's Global Assessment (sPGA), BSA improvement from baseline at weeks 12 and 24. Assessments were performed at screening, at baseline, and every 4 weeks thereafter throughout the study.	Percentage of patients with AEs.	1b
13.	Yu et al. (27)	Randomized trial, unclear blinding	30	PASI ≥10	Etanercept 50 mg once weekly plus oral methotrexate 7.5–15 mg per week	Etanercept 50 mg once weekly	24	24	PASI score, static Physician's Global Assessment (sPGA), Patient's Global Assessment (PtGA), Dermatology Life Quality Index (DLQI)	Percentage of patients with AEs.	2b
14.	Mease et al. (28)	Randomized, double-blind, placebo-controlled triple armed trial	851	3 tender joints and 3 swollen joints (based on 68- and 66-joint, and an active psoriatic skin lesion that was ≥2 cm in diameter).	Etanercept (target dose 50 mg) plus oral methotrexate (target dose 20 mg) given weekly.	Methotrexate (target dose 20 mg) plus subcutaneous placebo given weekly or subcutaneous etanercept (target dose 50 mg) plus oral placebo given weekly.	48	48	ACR20, Minimal Disease Activity (MDA) response, Leeds Dactylitis Index (LDI), static Physician's Global Assessment (sPGA).	Percentage of patients with AEs and SAEs/withdrawal because of AEs.	1b
15.	Baranauskaite et al. (29)	Open-label randomized trial	115	Psoriasis and psoriatic arthropathy	Infliximab 5 mg per kg infusions at weeks 0, 2, 6, and 14 plus methotrexate 15 mg per week	Methotrexate 15 mg per week	16	16	ACR20, ACR50 and ACR70 responses, PASI 75, PASI 90, EULAR response, physician and patient global assessment of disease activity, disease activity score in 28 joints (DAS28) scores, minimal disease activity (MDA)	Percentage of patients with AEs and SAEs/withdrawal because of AEs.	2b
16.	van Mens et al. (30)	Randomized, double-blind, placebo controlled trial	59	Patients meeting CASPAR criteria and current active disease, defined as the presence of at least three swollen and three tender joints.	Methotrexate 25 mg per week or as tolerated plus Golimumab 50 mg administered every 4 weeks	Methotrexate 25 mg per week or as tolerated plus placebo prefilled syringes administrated every 4 weeks	22	22	Disease Activity Score (DAS), MDA, ACR20/50/70 responses, Leeds Enthesitis Index, and Dermatology Life Quality Index (DLQI).	Percentage of patients with AEs and SAEs/withdrawal because of AEs.	2b
17.	Vieira-Sousa et al. (31)	Randomized, double-blind, placebo controlled trial	48	Classification for Psoriatic Arthritis criteria ≥1 digit with tender dactylitis and ≥1 other site of active inflammation (joints, enthesis, spine, skin, or nails).	Methotrexate 25 mg per week or as tolerated plus Golimumab 50 mg administrated every 4 weeks	Methotrexate 25 mg per week or as tolerated plus placebo prefilled syringes administrated every 4 weeks	24	24	Dactylitis Severity Score (DSS) DSS20, 50 or 70, Leeds Dactylitis Index (LDI) LDI20, 50 or 70, Enthesitis Index (LEI).	Percentage of patients with AEs.	2b
18.	Lee et al. (32)	Randomized, open labeled trial	60	≥10% BSA or PASI ≥10	Etanercept 25 mg biweekly plus acitretin 10 mg twice daily for 24 weeks	(i) Etanercept 50 mg biweekly for 12 weeks followed by etanercept 25 mg biweekly for 12 weeks; (ii) Acitretin 10 mg BID for 24 weeks.	24	24	PASI 75, PASI 50, clear/almost-clear by PGA	Percentage of patients with AEs	2b
19.	Gisondi et al. (33)	Randomized, controlled, investigator-blinded pilot trial	60	≥10% BSA or PASI ≥10	Etanercept 25 mg once weekly plus oral acitretin 0.4 mg per kg per day daily.	Etanercept 25 mg twice weekly subcutaneously; (ii) Acitretin 0.4 mg per kg per day daily in a single oral dose; and	24	24	PASI 75, PASI 50 and mean BSA reduction at week 24	Percentage of patients with AEs	2b
20.	van Bezooijen et al. (34)	Randomized controlled trial	33	PASI ≥10	Oral fumarates up to 4 × 215 mg plus Etanercept 2 × 50 mg/week for 12 weeks followed by 1 × 50 mg weekly from week 12 onwards	Etanercept at 2 × 50 mg/week for 12 weeks followed by etanercept to 1 × 50 mg weekly from week 12 onwards	48	48	PASI 75, PGA clear or almost clear	Percentage of patients with AEs	2b
21.	Tzaneva et al. (35)	Open-label randomized trial	30	≥10% BSA or PASI ≥10	Accelerated FAE dosing scheme with NB-UVB thrice weekly	Accelerated FAE	26	26	Mean PASI reduction, PASI 75, Mean, absolute and relative DLQI reduction	Percentage of patients with AEs	2b
22.	Prystowsky et al. (36)	Randomized, single blinded, placebo-controlled trial	19	>20% BSA	Calcitriol 0.5–2.0 μg per day plus NB-UVB four times weekly	Placebo plus NB-UVB four times weekly	5	NR	Mean change in PASI (scale, 0-16)	NR	2b
23.	Ezquerra et al. (37)	Open-label randomized trial	40	PASI ≥15	Acitretin at 25 mg per day plus calcitriol 0.25 μg per day	Acitretin at 25 mg per day	12	NR	Mean change in PASI	Percentage of patients with AEs	2b
24.	Mittal et al. (38)	Randomized, double-blind, placebo-controlled trial	41	>20% BSA	Acitretin at 25 mg per day plus pioglitazone, Hydrochloride at 15 mg per day	Acitretin at 25 mg per day plus placebo	12	12	PASI 75, PGA of clear or almost clear, mean change in PASI, withdrawal because of lack of efficacy	Percentage of patients with AEs, withdrawal because of AEs	2b
25.	el-Mofty et al. (39)	Randomized trial, unclear masking	16	>25% BSA	Sulfasalazine, 2 gm per day plus Pentoxifylline 1,200 mg per day	Methotrexate, 25 mg per week	8	NR	Mean change in PASI, Withdrawal because of lack of efficacy	Percentage of patients with AEs	2b

*PUVA, Psoralen and Ultra Violet A; UVB, Ultra Violet B; NB-UVB, Narrow Band Ultra Violet B; BSA, Body Surface Area; PASI, Psoriasis Area and Severity Index; AE, Adverse Events; SAE, Serious Adverse Events; BMI, Body Mass Index; DLQI, Dermatology Life Quality Index; PGA, Physician's Global Assessment; sPGA, static Physician's Global Assessment; PtGA, Patient's Global Assessment; ACR 20/50/70, American College of Rheumatology 20/50//70; MDA response, Minimal Disease Activity response; LDI 20/50/70, Leeds Dactylitis Index 20/50/70; EULAR response, European League Against Rheumatism response; DAS28 scores, Disease Activity Score in 28 joints; LSI, Leeds Enthesitis Index*.

**Table 2:** Summary of levels of evidence and strength of recommendations.

**S. no**.	**Drug combinations**	**Highest levels of evidence on efficacy**	**Recommendations for combination on basis of evidence**
1	UVB/PUVA + Acitretin (15, 16)	2b	B
2	Etanercept + NB-UVB (17–20)	1b, 2b	A
3	Adalimumab + NB-UVB (40, 41)	2b, 4	B
4	Ustekinumab + NB-UVB (21)	2b	B
5	Methotrexate + NB-UVB (22–24)	1b, 2b	A
6	Etanercept + Methotrexate (25–28)	1b, 2b	A
7	Infliximab + Methotrexate (29)	2b	B
8	Golimumab + Methotrexate (30, 31)	2b	B
9	Etanercept + Acitretin (32, 33)	2b	B
10	Apremilast + NB-UVB (42)	4	C
11	Apremilast + Secukinumab (43, 44)	4	C
12	Etanercept + Fumarates (34)	2b	B
13	Fumarates + NB-UVB (35)	2b	B
14	Calcitriol (oral) + Acitretin (37)	2b	B
15	Hydroxyurea + Acitretin (45)	4	C

*Levels of evidence: 1a, Systematic review of (homogeneous) RCTs; 1b, Individual RCTs (with narrow confidence intervals); 2a, Systematic review of (homogeneous) cohort studies of “exposed” and “unexposed” subjects; 2b, Individual cohort study/low-quality RCTs; 3a, Systematic review of (homogeneous) case-control studies; 3b, Individual case-control studies; 4, Case series, low-quality cohort or case-control studies; 5, Expert opinions based on non-systematic reviews of results or mechanistic studies*.

*Strength of recommendations: A, Good evidence to support a recommendation for use; B, Moderate evidence to support a recommendation for use; C, Poor evidence to support a recommendation*.

### Discussion and Recommendations

Literature search yielded 25 RCTs combining different agents to treat psoriasis and/or psoriatic arthropathy. Most of the studies involved combinations with Narrow Band Ultraviolet B (NB-UVB) /Psoralen with Ultraviolet A (PUVA) or methotrexate.

### NB-UVB/PUVA and Acitretin

There are 02 RCTs involving acitretin and UVB/PUVA by Tanew et al. (15) and Lowe et al. (16). The former found that the cumulative PUVA dose required for complete clearance in PUVA-acitretin group was 58.7 ± 17.9 J/cm^2^ whereas in PUVA-placebo group was 101.5 ± 15.8 J/cm^2^. In RCT by Lowe et al. (16), 14 participants in the UVB-acitretin group took a total of 873 min of UVB exposure for complete clearance as compared to a significantly higher time- 1,236 min in the UVB-placebo group (*n* = 15). At the end of 12 weeks, the mean PASI ± SD in acitretin + UVB group reduced significantly from 8.83 ± 1.8 to 2.27 ± 1.04 (p < 0.01), whereas it reduced from 9.75 ± 2.34 to 6.36 ± 3.07 in placebo + UVB group. Both the RCTs concluded that adding UVB/PUVA to acitretin achieves greater as well as faster clearance than either placebo- UVB/PUVA or acitretin alone. Clinical adverse effects of added acitretin in both the studies were generally well-tolerated and similar to previous studies in treatment of psoriasis with acitretin (46–48).

### Recommendation

We recommend combining these two modalities when patients do not respond to either one of the two. In addition to increased efficacy, the combination allows reduction of cumulative dose of UVB/PUVA. Also important is the prevention of non-melanoma skin cancers by acitretin which may be caused by long term UVB/PUVA (49, 50).

### NB-UVB With TNFα Inhibitors

Etanercept with NB-UVB combination has been evaluated by Lynde et al. (17), Park et al. (18), Calzavara-Pinton et al. (19), and Gambichler et al. (20). Lynde et al. (17) concluded that addition of NB-UVB to etanercept did not significantly improve the overall clinical response except for a subset of patients with high adherence to NB-UVB without increasing the adverse effects significantly. Park et al. (18) studied combination of etanercept and NB-UVB in obese patients. They concluded that the combination has a similar efficacy to etanercept monotherapy even in the setting of obesity. However, Calzavara-Pinton et al. (19) who performed an intra-individual RCT in receiving etanercept and a randomized half of the body with NB-UVB for found that The PSI (Psoriasis Severity Index) scores of non-irradiated control lesions were 6.4 ± 2.3 and 5.8 ± 2.5 (*p* = not significant) before and after the treatment respectively, whereas the PSI of irradiated psoriatic plaques were 6.3 ± 2.3 and 0.5 ± 0.8 (*p* < 0.05). In the combination group, the mean PASI ± SD value reduced from 16.2 ± 9.2 to 2.4 ± 2.8 in 12 weeks. The patients received 14.6 ± 3.3 exposures resulting in a cumulative dose of 8.4 ± 4.2 J cm^−2^. While the combined treatment was always well-tolerated, it was aimed at short duration of NB-UVB therapy for faster clearance to avoid long term adverse effects. It also may help reduce total doses as well as cost of etanercept therapy. Calzavara-Pinton et al. (19) inferred that the combination is more effective than each therapy alone in the treatment of moderate-to-severe plaque psoriasis, and is well-tolerated. In an intra-individual RCT by Gambichler et al. (20) (*n* = 14) the relative M-PASI (modified-PASI) reduction of etanercept alone treated sites after 6-weeks was 53.7 ± 36.9%, whereas etanercept plus NB-UVB combination treated sites resulted in a significantly higher relative M-PASI reduction of 64.7 ± 27.8% (*P* = 0.011, 95% CI −19 to −3%) concluding that etanercept combined with NB-UVB is more effective than etanercept monotherapy at 6 weeks. Similarly, in an another intra-individual RCT by Wolf et al. (40) consisting of 04 participants who were followed up for 06 weeks concluded that adding thrice weekly NB-UVB to 40 mg bi-weekly adalimumab reduced mean PASI from 14.8 to 2.0 on UV-irradiated body halves vs. 6.9 on non-irradiated body halves (95% confidence interval, 0.4–9.4) accelerating the clearance of psoriatic lesions with no significant adverse effects. Bagel (41) performed a 24-week single-arm open-label study in 20 adults with moderate to severe psoriasis who received bi-weekly adalimumab 40 mg and thrice weekly NB-UVB phototherapy for 12 weeks and followed up for another 12 weeks. The mean baseline scores of patients were 17.0 for PASI, 21.2 for BSA (Body Surface Area) and 3.5 for PGA (Physicians Global Assessment). At the end of treatment at week 12, 19 (95%) patients achieved PASI-75, 15 (75%) PASI-90 and 11 (55%) achieved PASI-100. Seventeen (85%) were clear or almost clear (PGA score =1). Mean baseline PASI, BSA, and PGA scores improved by 95, 93, and 80%, respectively. Moreover, the improvement was sustained through the end of follow up period at week 24 without any serious adverse events. Although none of the studies combining TNFα blockers and NB-UVB reported any major adverse effects, concerns were shown regarding the long-term effects of combining TNFα blockers with NB-UVB- especially malignancy.

### Recommendation

As the implication of malignancy in treatment with TNF-α blockers alone or in combination with NB-UVB complex with levels of TNF-α having varied effects on tumoral growth, (51) we recommend to restrict this highly effective combination for short duration up to 24 weeks, to obtain a quicker response and to avoid long-term complications (52–54). European Academy of Dermatology and Venereology (EADV) guidelines on management of psoriasis mention that TNFα blockers and NB-UVB may or may not be combined and it is not as strict a contraindication as cyclosporine with NB-UVB (55).

### NB-UVB and IL12/23 Inhibitor

There is only a single intra-individual RCT combining injection ustekinumab at 45/90 mg 4 weeks apart and thrice weekly 311-nm UVB by Wolf et al. (21) in 10 patients. At baseline, the mean PASI was similar in both irradiated and unirradiated body halves (13.6 vs. 13.3). At 6 weeks, PASI was significantly lower on irradiated body halves (2.5 vs. 6.1), (95% confidence interval 1.3–5). PASI 75 was achieved significantly more often on UV-irradiated body halves than on un-irradiated ones [7/9 patients (78%) vs. 1/9 (11%)]. They concluded that treatment with NB-UVB accelerates the clearance of psoriatic lesions at week 6 as well as at week 12 in ustekinumab-treated patients without increase in incidence of severe adverse effects.

### Recommendation

No specific recommendation could be offered as there is limited review of this combination. However, in patients on ustekinumab with a poor response NB-UVB may be added as it has a good safety profile.

### NB-UVB and Methotrexate

03 studies combining methotrexate and NB-UVB met the criteria to be included in our review- Mahajan et al. (22), Asawanonda et al. (23), and Al-Hamamy et al. (24). Mahajan et al. (22) combined oral methotrexate at 0.5 mg/kg once weekly [maximum of 30 mg/week and thrice weekly NB-UVB and compared it with placebo plus NB-UVB for a duration of 12 weeks. PASI 75 was attained in 19/20 patients in the combination group versus 14/20 patients in NB-UVB plus placebo group (*p* = 0.04)]. PASI 75 was achieved in 7.57 ± 3.09 weeks (4–16) in the combination group and 11.42 ± 4.98 weeks (6–20) in NB-UVB + placebo group (*p* < 0.006). The mean number of NB-UVB sessions to which the patients were exposed were 17.47 ± 6.62 (10–35) in the combination group and 35.72 ± 17.05 (16–6) in NB-UVB + placebo group (*p* < 0.0001). Mean NBUVB dose for achieving PASI 75 was 9.14 ± 5.39 J/cm^2^ (3.34–20.84) in the combination group as compared with 25.99 ± 18.55 J/cm^2^ in NB-UVB + placebo group (*p* < 0.001). Asawanonda et al. (23) showed that the median time to clear psoriasis in the former group was 4 weeks, which was significantly less than that the latter. Ten of 11 patients on combination of methotrexate and NBUVB achieved PASI 90 compared with only 5/13 in the placebo/ NB-UVB group (*p* < 0.0001). The mean cumulative dose in methotrexate/NB-UVB group was 26.92 ± 15.54 J/cm^2^, as compared to 59.25 ± 16.71 J/cm^2^ (*p* = 0.002) in the placebo/NBUVB group. Al-Hamamy et al. (24) compared the combination of methotrexate with NB-UVB, methotrexate alone and NB-UVB alone and found no statistically significant difference in the number of patients achieving PASI 90 between the three groups in six months of treatment. However, the mean number of weeks required for achieving clearance was 6.11 ± 1.28 weeks in combination group and 11.42 ± 2.36 weeks in NB-UVB group, while 20.87 ± 4.21 weeks in methotrexate group (*p* < 0.0001). The mean number of NBUVB sessions to which the patients were exposed was 17.86 ± 3.74 in combination group and 33.51±6.90 in NB-UVB group (*p* < 0.0001). The mean total cumulative dose of NBUVB phototherapy for achieving clearance was 12.13 ± 4.02 J/cm^2^ in the combination group; compared with 34.48 ± 13.13 J/cm^2^ in NB-UVB group (*p* < 0.0001). All 03 RCTs combining methotrexate with NB-UVB concluded that the mean time to achieve reduction in PASI 75 was significantly less in the combined group as against those treated only with NB-UVB and addition of methotrexate to NB-UVB rapidly clears psoriatic lesions without any significant adverse effects.

### Recommendation

We recommend combining NB-UVB with methotrexate for faster clearance of lesions. However, either may be discontinued after achieving PASI 75 and the other continued for maintenance therapy the duration of which shall be dictated by the disease burden.

### TNFα Inhibitors and Methotrexate

The following RCTs combining TNFα blockers with methotrexate met the inclusion criteria- Zachariae et al. (25), Gottlieb et al. (26), Yu et al. (27), Mease et al. (28), Baranauskaite et al. (29), van Mens et al. (30), and Vieira-Sousa et al. (31) who compared the combination of TNFα blockers with methotrexate with either TNFα blockers alone or methotrexate alone. Zachariae et al. (25) randomized 60 patients who were already on methotrexate for at least 03 months into two groups receiving etanercept-methotrexate combination and etanercept with tapering and stopping methotrexate and noted significantly more number of patients achieving PASI 75 as well as significantly lower mean PASI scores at both 12 and 24 weeks in the combination group compared with etanercept alone with similar AEs for both groups, an effect that was maintained until the end of the study. Gottlieb et al. (26) studied 478 patients combining weekly methotrexate to patients who were already on etanercept since 24 weeks to the treatment group against placebo in control group. The percentage of patients achieving PASI 75 was significantly higher at week 24 for the combination therapy group (77.3%) compared with the monotherapy group (60.3%; *p* < 0.0001). Overall, 74.9% of patients in the combination group experienced AEs compared with 59.8% in the monotherapy group. Withdrawals due to AEs were infrequent in both groups [combination, *n* = 10 (4.2%); monotherapy, *n* = 6 (2.5%)], and none of the AEs leading to withdrawal was considered to be serious or infectious. They concluded that addition of methotrexate to etanercept was more effective than etanercept monotherapy in patients with moderate to severe plaque psoriasis with acceptable tolerability. Yu et al. (27) compared similar treatment arms as above but started administering the combination from baseline and followed up subjects for 24 weeks. They found no significant change in the PASI score from baseline to 24 weeks. However, both sPGA (static Physician's Global Assessment) and PtGA (Patient's Global Assessment) scores were significant (*p* < 0.05). Adverse effects were reported in 60% of patients in the combination group and in 33% in the monotherapy group. None of the adverse effects were serious enough to discontinue treatment. Mease et al. (28) performed a triple arm study consisting of 851 patients of psoriatic arthropathy randomized to oral methotrexate 20 mg plus placebo weekly, etanercept 50 mg plus placebo weekly, or etanercept 50 mg plus oral methotrexate 20 mg weekly. ACR20 (American College of Rheumatology 20) criteria and MDA (Minimal Disease Activity) responses at week 24 were significantly greater for etanercept monotherapy vs. methotrexate monotherapy (ACR20: 60.9 vs. 50.7% [*p* = 0.029]; MDA: 35.9 vs. 22.9% [*p* = 0.005]) and for combination therapy vs. methotrexate monotherapy (ACR20: 65.0 vs. 50.7% [*p* = 0.005]; MDA: 35.7 vs. 22.9% [*p* = 0.005]). Many patients in this trial had a moderate to severe level of psoriasis as assessed by BSA (Body Surface Area). Results from the dermatologic endpoints showed that etanercept and methotrexate had good efficacy, with a suggestion that the combination arm had slightly greater efficacy than either of the monotherapy arms for improved BSA. They concluded that etanercept monotherapy and combination therapy showed greater efficacy than methotrexate monotherapy in ACR, MDA, and BSA responses and radiographic progression. However, combining methotrexate and etanercept did not improve etanercept efficacy in either PsA or psoriasis. Baranauskaite et al. (29) combined infliximab at 5 mg per kg infusions at 0, 2, 6, and 14 weeks and methotrexate at 15 mg per week vs. methotrexate alone for a period of 16 weeks. 86.3% of patients receiving combination and 66.7% of those receiving methotrexate alone achieved an ACR20 response (*p* < 0.02). While 97.1% of patients receiving infliximab plus methotrexate achieved PASI 75, the figure was 54.3% in patients receiving methotrexate alone (*p* < 0.0001). They demonstrated significantly greater ACR 20 response rates and PASI 75 improvement in the combination group and was generally well-tolerated. A double-blind RCT measuring end points in psoriatic arthritis by van Mens et al. (30) studied combination of methotrexate 15–25 mg per week and subcutaneous injections of golimumab at 50 mg per month with that of methotrexate and placebo and found that Disease Activity Score (DAS) remission at week 22 was almost doubled (21/26;81%) in methotrexate plus golimumab group vs. methotrexate alone (10/24; 42%) (*p* = 0.004). Also the patients belonging to the combination group reached an MDA (Minimal Disease Activity) in 21/26 (81%) vs. 7/24 (29%) in the methotrexate arm (*p* < 0.001). An ACR 20/50/70 response was achieved by, respectively, 85, 81, and 58% in the combination arm vs. 58, 33, and 13% in the methotrexate arm (*p* = 0.039, *p* = 0.001, and *p* = 0.001, respectively). The most frequent adverse effect was nausea and occurred in similar incidences in both treatment arms and considered to be treatment related but was not severe enough to discontinue treatment. Likewise, a double-blind RCT by Vieira-Sousa et al. (31) comparing similar doses of golimumab plus methotrexate vs. placebo plus methotrexate in dactylitis in psoriatic arthropathy concluded that the combination of golimumab and methotrexate was superior to methotrexate alone in reducing Dactylitis Severity Score (DSS) and Leeds Dactylitis Index (LDI) with comparable incidence of adverse effects between treatment arms. All patients had active dactylitis at baseline, with a median baseline DSS of 6 in both arms. The patients treated with golimumab/methotrexate exhibited significantly greater improvements by DSS at week 24 (median change of 5) relative to the placebo/methotrexate group (median change of 2) (*p* = 0.026), and as early as 12 weeks (*p* = 0.004). The proportion of DSS50 (Dactylitis Severity Score 50) and DSS70 (Dactylitis Severity Score 70) responders at week 24 were also significantly higher for patients treated with golimumab/methotrexate (DSS50: *p* = 0.005, DSS70: *p* = 0.010) Endpoints to measure cutaneous efficacy like PASI, BSA and skin-related quality of life (Dermatology Life Quality Index) improved in both groups at week 24 but difference in both treatment groups was not significant. 102 adverse events were reported during study period, with similar incidence between the treatment arms and mostly of mild to moderate severity. According to systematic review by Hsu et al. (56), there are 06 studies measuring anti-drug antibodies in etanercept and its possible effect on drug efficacy- they found the prevalence of anti- etanercept antibodies (AEA) ranging from 0 (57) to 18.3% (58) in psoriasis, and none of which had significant effect on treatment efficacy (56). Similarly, 10 studies proved prevalence of anti-infliximab antibodies (AIA) ranging from 5.4 (59) to 43.6% (60) with most of these studies showing significant decreased mean PASI scores and greater loss of clinical response when compared to AIA-negative patients (56). A study by Adisen et al. (61) with five patients of psoriasis who developed AIA, determined that AIA positivity disappeared after 8 weeks of combined methotrexate pulse therapy, ranging from 5 to 15 mg/week. Six studies assessed for Anti-Ustekinumab Antibody (AUA) formation in patients with moderate-to severe psoriasis showed ranges from 3.8 (62) to 5.4% (63) in psoriasis (56). But their clinical significance on treatment response is yet to be evaluated (56).

### Recommendation

We recommend combining TNFα blockers with methotrexate in moderate to severe psoriatic disease especially while using infliximab. Poor response to etanercept alone at lower doses as elaborated below necessitates an additional drug, methotrexate being a good option.

### TNFα Inhibitors With Cyclosporine

Atzeni et al. (64) who performed an RCT comparing etanercept plus cyclosporine with etanercept plus methotrexate show similar efficacy in reducing DAS28 (Disease Activity Score 28) in patients with moderate/severe psoriatic arthropathy and peripheral arthritis, but former combination was more efficacious in reducing psoriatic skin involvement. PASI 50 and PASI 75 scores were achieved by 88 and 53%, respectively, in the patients in etanercept plus cyclosporin group, and 73 and 32%, respectively, in the patients in etanercept plus methotrexate group (*p* < 0.05). There was no significant difference in serious adverse events between the two treatment groups.

### Recommendation

We recommend TNFα blockers with cyclosporine in moderate to severe psoriasis with arthropathy for rapid remission, however side effects limit the duration of treatment with cyclosporine and sequential therapy with methotrexate is recommended.

### TNFα Inhibitors With Acitretin

Lee et al. (32) randomized 60 subjects into three treatment arms- ETN-ETN (etanercept-etanercept), ETN-ACT (etanercept-acitretin), and ACT (acitretin). The median time to achieve PASI 75 for patients in the ETN–ETN arm was 126 days vs. 146 days for patients in the ETN-ACT arm. The median time to achieve PASI 50 was same in ETN–ETN and ETN-ACT arms (56 days) and much shorter than for patients in ACT arm (126 days). The difference was statistically significant among the three treatment arms (PASI 75: *p* = 0.0448 and PASI 50: *p* = 0.0033). Lee et al. (32) proved that the combination is more effective than acitretin alone without increase in adverse effects. In another study with similar treatment arms Gisondi et al. (33) randomized 60 patients into three groups to receive etanercept 25 mg twice weekly; acitretin 0.4 mg per kg daily; and etanercept 25 mg once weekly plus oral acitretin 0.4 mg per kg daily. PASI 75 response at week 24, was achieved by 10 of 22 patients (45%) in the etanercept group, six of 20 (30%) in the acitretin group and eight of 18 (44%) patients with etanercept plus acitretin group (*P* = 0.001 for both etanercept groups compared with acitretin alone). PASI 50 response at week 24 too showed similar significant results (*P* = 0.001 for both etanercept groups compared with acitretin alone).

### Recommendation

Etanercept 25 mg twice weekly with acitretin is a superior option to acitretin alone. We recommend addition of acitretin to etanercept dose of 25 mg twice weekly before considering a higher dose of etanercept 50 mg twice weekly.

### Apremilast Combinations

Apremilast, a PDE4 inhibitor has minimal immunosuppressive effects when compared to biologics. There are no RCTs combining apremilast with any other drug. However, case series and retrospective studies have suggested that combination of apremilast with other drugs and biologics like methotrexate, acitretin, cyclosporin, secukinumab, etanercept, adalimumab, ixekizumab, and ustekinumab have been effective, look promising and may be exercised to reduce adverse effects of either of two.

In an open-labeled prospective study combining apremilast 30 mg twice daily and increasing doses of NB-UVB three times per week for 12 weeks. 73% (16 of 22 completers) achieved a PASI 75 response at week 12. The most commonly reported adverse events were mild and moderate first-degree burns related to NB-UVB (*n* = 11 [38%] patients). Bagel et al. (42) concluded that the combination provided a new treatment option without any increased adverse effects. Both Sacchelli et al. (43) and De et al. (44) published case series and case reports combining apremilast with secukinumab and found improvement in PASI scores. Metyas et al. (65) and Takamura et al. (66) performed retrospective studies reporting the efficacy of apremilast in combination with any other biologics and inferred that apremilast can be safely combined with all biologic agents in patients with plaque psoriasis or psoriatic arthritis not responding adequately to biologics alone. Another retrospective study by AbuHilal et al. (67) studied the combination of apremilast with other biologics as well as conventional drugs like methotrexate, acitretin, cyclosporine with similar conclusion.

### Recommendation

We recommend apremilast 30 mg twice daily and NB-UVB as a combination modality not responding or minimally responding to either of the two. The combination of apremilast and a biologic may be a safe, useful treatment option for managing patients with psoriasis showing biologic fatigue, but not as a routine. However, large scale studies with higher level of evidence like RCTs are needed in future.

### Miscellaneous Combination Therapies

RCTs combining less used unconventional drugs in psoriasis included in this review dealt with fumaric acid esters (FAEs) calcitriol, sulfasalazine, pentoxifylline, and pioglitazone with conventional modes of therapy. An exploratory RCT by Bezooijen et al. (34) combining etanercept 50 mg twice weekly for 12 weeks followed by once weekly for another 12 weeks with oral fumarates 215 mg four times daily for the whole period vs. etanercept alone found out that the reduction in PASI score per week for the combination therapy was 5.97% (95% confidence interval, CI: 5.08–6.85) and in the monotherapy group 4.76% (95% CI: 3.57–5.93; *p* = 0.11). They concluded that combination therapy caused quicker improvement in PASI 75 in first 24 weeks although difference in the PASI score between the two groups was statistically insignificant but with satisfactory tolerability. In an another RCT by Tzaneva et al. (35), an increasing dose of FAEs was combined with NB-UVB. At 26 weeks of treatment, the median baseline PASI of 15.4 [interquartile range (IQR) 11.7–21.0] was reduced to 2.8 (IQR 1.6–4.8) in the combination group and from 14.0 (IQR 12.5–15.1) to 9.0 (IQR 6.5–12.1) in the FAE group, respectively. The mean absolute and relative reduction in PASI was significantly greater in the combination group (10 and 69%) compared with patients receiving only FAE (5 and 36%) (*p* = 0.016). Side-effects related to FAE were mainly mild gastrointestinal complaints reported by 12/16 patients (75%) in the monotherapy group and 3/14 patients (21%) in the combination group. These were abdominal pain, nausea, flatulence, diarrhea that occurred at the beginning of treatment, were dose-dependent and improved after a temporary dose reduction. They found an accelerated as well as augmented response improving the quality of life in the patients with combination therapy as compared with fumaric acid esters monotherapy with no increase in adverse effects in the combination group.

A single blinded, placebo-controlled trial combining calcitriol 0.5–2.0 μg per day plus NB-UVB against NB-UVB alone by Prystowsky et al. (36) concluded that there was no added benefit to treatment when oral calcitriol was administered with phototherapy. Our search yielded only a single RCT combining acitretin and calcitriol- Ezquerra et al. (37) who compared the combination with acitretin alone. Initial PASI of 26.90 reduced to 13.3 in acitretin alone group; whereas it reduced from 28.35 to 10.3 in acitretin+calcitriol combination group which was statistically significant (*p* < 0.05). A double-blind RCT by Mittal et al. (38) compared combination of acitretin plus pioglitazone hydrochloride with acitretin alone. The percentage of reduction in the PASI score from baseline to 12 weeks of treatment was 64.2% (95% CI, 49.2–79.3%) in the combination group compared with 51.7% (95% CI, 38.7–64.7%) in the acitretin plus placebo group (*p* = 0.04). The adverse effects in both the groups were mild to moderate and were comparable. el-Mofty et al. (39) conducted a quadri-armed RCT comparing the combination of sulfasalazine and pentoxifylline to methotrexate alone (active control group), sulfasalazine alone and pentoxifylline alone and concluded that combination of sulfasalazine and pentoxifylline though effective than when used alone, is not as effective as methotrexate, may be promising and tried because they present as safer and well-tolerated alternatives to methotrexate. There are no RCTs on hydroxyurea in psoriasis. Hydroxyurea becomes one of the drugs of choice in settings of psoriasis in HIV, where not only it helps in controlling psoriasis, but also in controlling viral loads especially when combined with didanosine (NRTI) (68, 69). In a retrospective study, Narang et al. (45) combined lower doses of hydroxyurea 1 g daily with acitretin 25 mg daily for the management of refractory cases and found them to be superior to either to hydroxyurea and acitretin alone as found in previous studies. Combining acitretin with hydroxyurea may theoretically reduce the risk of non-melanoma skin cancers and actinic keratosis, which are rare but serious adverse effect of hydroxyurea (49, 70). Methotrexate too have been combined with hydroxyurea in lower doses (5–10 mg/week and 500 mg per day, respectively) to good effect with no increase in adverse effects of either of the two (71). Though theoretically both the drugs may cause GI intolerance and myelosuppression, they were not found in the study.

### Recommendation

We recommend combining hydroxyurea and acitretin in recalcitrant cases of psoriasis not responding to conventional stand-alone drugs. This combination also may be used in HIV where both the drugs do not cause immune suppression with added benefit of anti-viral action of hydroxyurea.

Combining methotrexate with hydroxurea in lower doses may help reducing dose-dependent or cumulative toxic effects of either of the two.

Our search for combinations comprising relatively newer drugs like guselkumab, tildrakizumab, certolizumab pegol, and tofacitinib yielded no results and provide gap in research with a massive potential.

## Conclusion

Combining newer therapies with conventional ones is a promising prospect to manage difficult to treat psoriasis. Combining drugs when suited to patients needs can enhance efficacy, achieve remission, while reducing adverse effects. With available evidence, there are limited options with highest level of evidence and hence recommendation. Due to a smaller number of studies in combination of drugs, research providing more high-quality evidence is required.

## Data Availability Statement

The original contributions presented in the study are included in the article/supplementary material, further inquiries can be directed to the corresponding author.

## Author Contributions

SA: conception and design, acquisition of data, literature search, analysis and interpretation of data, drafting the manuscript, and revising it. PD and GA: acquisition of data, literature search, analysis and interpretation of data, drafting the manuscript, and revising it. All authors contributed to the article and approved the submitted version.

## Conflict of Interest

The authors declare that the research was conducted in the absence of any commercial or financial relationships that could be construed as a potential conflict of interest.

## Publisher's Note

All claims expressed in this article are solely those of the authors and do not necessarily represent those of their affiliated organizations, or those of the publisher, the editors and the reviewers. Any product that may be evaluated in this article, or claim that may be made by its manufacturer, is not guaranteed or endorsed by the publisher.
